# N Management of European Grasslands: Can the Exchange of Gaseous N Species Be Influenced at the Operational Level?

**DOI:** 10.1100/tsw.2001.373

**Published:** 2001-11-30

**Authors:** P. Calanca, A. Neftel, J. Fuhrer

**Affiliations:** Swiss Federal Research Station for Agroecology and Agriculture, Liebefeld, Berne, Switzerland

**Keywords:** grassland ecosystems, management, emissions, nitrous oxide, ecosystem models

## Abstract

Grassland ecosystems can be regarded as biochemical reactors in which large amounts of organic nitrogen (N) are converted into inorganic N, and vice versa. If managed in a sustainable manner, grasslands should operate in a quasi steady state, characterized by an almost perfect balance between total N input and output. As a consequence, the exchange of gaseous N species (NH_3_, NO, NO_2_, N_2_O, and N_2_) between grasslands and the atmosphere is very small compared to the total N turnover. In this study, the effects of two management options (mowing and fertilization) on production and emission of nitrous oxide (N_2_O) from a grass/clover crop were examined on the basis of observations and model results referring to an experiment carried out on the Swiss Plateau in late summer of 2000. It was found that production and emission of N_2_O induced by mowing were of the same order of magnitude as those brought about by fertilization, suggesting a possible transfer of N from clover to the soil after defoliation. Emissions were strongly modulated by precipitation on time scales ranging from 1 day to 1 week. This indicates that effective control of N_2_O emissions through management on a day-to-day basis requires reliable medium-range weather forecasts. Model calculations were not able to reproduce essential characteristics of the emissions. The model slightly overestimated the background emissions, but severely underestimated the emission peaks following fertilizer application, and largely failed to reproduce emission induced by mowing. Shortfalls in the model used for this study were found in relation to the description of soil-water fluxes, soil organic matter, and the physiology of clover.

## INTRODUCTION

Gaseous N species are of paramount importance for the chemistry of the atmosphere and are crucial in the context of climate change[1]. Due to human activities, their atmospheric concentrations are drastically increasing. Agriculture substantially contributes to the anthropogenic emission of nitrous oxide ($N_2O$) and ammonia ($NH_3$), and needs to be taken into account in the discussion of mitigation policies[2]. In this context, the key question is whether or not management of agricultural ecosystems alone can exert an active control on the exchange of gaseous N-species, specifically $N_2O$.

The classification of regulation strategies at the farm level[3] (Fig. 1) was discussed at the International Workshop on Dissipation of N from the Human N-Cycle, and Its Role in Present and Future $N_2O$ Emissions to the Atmosphere[4]. It was pointed out that, although regulations at the strategic and/or tactical level are most suitable, large benefits can be obtained at the operational level — that is, on a day-to-day basis. This is the level considered in the present paper.

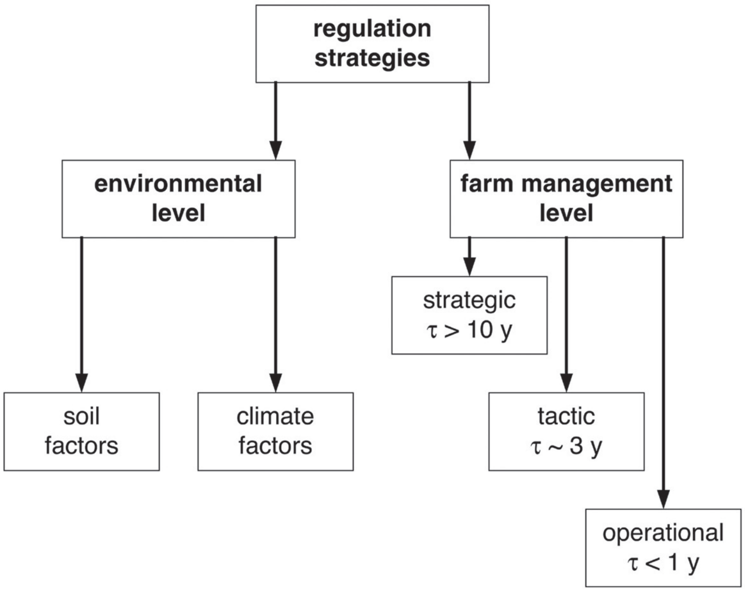

**FIGURE 1.** Classification of regulation strategies according to Oenema et al.[3]. Note the categorization of regulations at the farm management level according to the time scale of the expected effects. τ is the time scale.

An effective management at the operational level (including analysis, options, decision, implementation, and monitoring; see Fig. 1 in Mosier et al.[4]) requires the integration of all relevant physical and chemical processes regulating the emission of $N_2O$, combined with reliable medium-range forecasts of the environmental conditions. As for the understanding of the dynamics of agricultural ecosystems, in general, and of grassland ecosystems, in particular, both comprehensive field experiments and modeling activities are necessary. Despite significant advances in observational techniques during the last decade[5,6,7], process-oriented models remain an indispensable tool for the investigation of interactions and feedbacks (Fig. 2).

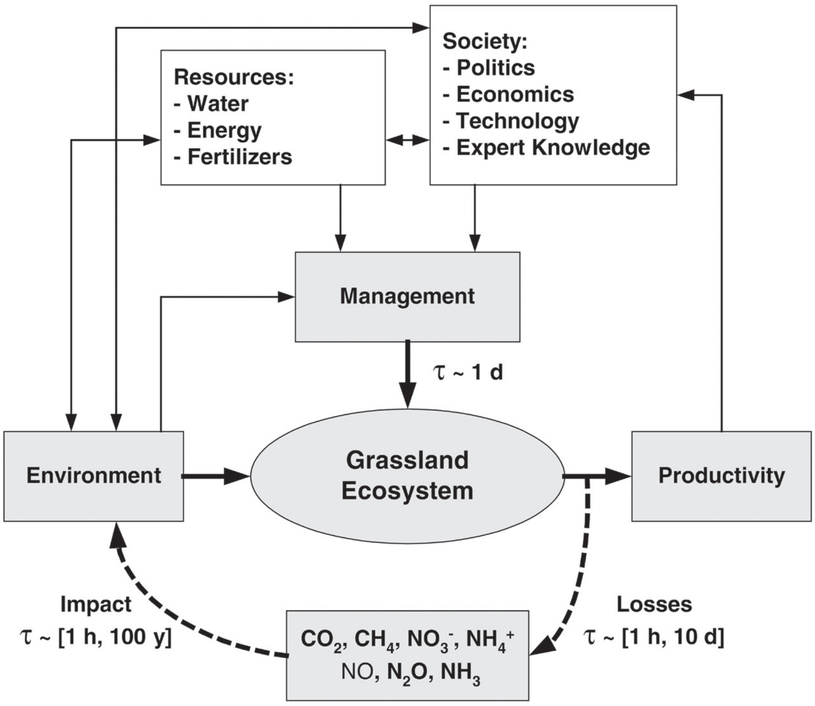

**FIGURE 2.** Management at the operational level is determined by the availability of resources and by societal needs. Together with the environment, with which it interacts, management sets the boundary conditions for the operation of grasslands, and determines therefore the productivity of the systems but also the losses. Ecosystem models typically consider only those elements outlined in grey and (inter)actions drawn in bold style. τ is the turnover time.

In practice, the complexity of the environmental factors and their interactions with the management limit the performance of process-oriented ecosystem models[8]. Most generally, difficulties arise because of the intrinsic characteristics of the N cycle. For European grasslands, for instance[9,10], the typical annual N turnover is of the order of 1000 kg N $ha^{-1}$ $year^{-1}$, whereas net fluxes of $NH_3$ and $N_2O$ are only of the order of 1 to 10 kg N $ha^{-1}$ $year^{-1}$. This means that reliable calculations of the net exchange are possible only provided that the accuracy of the calculated turnover is very high.

A few characteristic shortfalls of currently available ecosystem models were revealed by an intercomparison of four state-of-the-art models carried out in the framework of the U.S. Trace Gas Network program[11]. Specifically, the results showed that: (1) annual fluxes of N species were simulated at best within a factor of 2; (2) the simulation of $NH_3$ and nitric oxide (NO) was more problematic than the simulation of $N_2O$, but that (3) large errors occurred also in the simulation of individual $N_2O$-emissions peaks; (4) the models were not able to capture the effects of different types of fertilizers; (5) the models could not reproduce $N_2O$ pulses during brief winter thaws; and (6) the accurate simulation of soil moisture was a necessary prerequisite for the correct simulation of $N_2O$ emissions.

In our study, we apply a grassland ecosystem model to a case study of $N_2O$ production and release with particular consideration of the influence of precipitation and of two types of management. We aim to discuss: (1) the qualitative effects of the two types of management; (2) the impact of environmental factors; and (3) the capability of this specific ecosystem model to simulate $N_2O$ emission and the effects of management.

The data refer to an experiment carried out in the late summer of 2000 at Kerzersmoos (46°59′42″N, 7°11′02″E, 436 m.a.s.l.), a site located on the Swiss Plateau, in a flat rural area of the Seeland, 20 km northwest of the city of Bern. The site was characterized by a mollic gleysol, with remains of an organic layer at 65- to 72-cm depth, and a pH between 7.5 and 8.0. The sward was a mixture of grass and clover, with a clover fraction of approx. 30%[10]. The field was divided in two plots. Prior to the experiment, one plot (Kerzers West, low N) was treated with about half the amount of mineral N applied to the other plot (Kerzers East, high N). During the experiment, both plots were mown on August 21st. On August 29th, Kerzers East received an additional 20 kg N $ha^{-1}$ in the form of mineral fertilizer.

## THE PASTURE SIMULATION MODEL PASIM

The Pasture Simulation Model PaSim[12,13,14] is a process-oriented ecosystem model that simulates dry matter production and fluxes of C, N, water, and energy in permanent grassland ecosystems with a high temporal resolution. PaSim consists of submodels for plant growth, microclimate, soil biology, and soil physics. It is driven by hourly or daily weather data. Site-specific model parameters include the N input from mineral and/or organic fertilizers and atmospheric deposition, the fractional clover content of the grass/clover-mixture, the depth of the main rooting zone, and soil physical parameters, including soil texture and bulk density. The soil is divided into up to six layers.

Soil biology is basically described as in the CENTURY model[15]. Organic C and N are partitioned among five compartments, each characterized by its own turnover time but otherwise homogeneously distributed over the whole soil. The description of the N cycle includes mineralization, immobilization, nitrification, denitrification, plant uptake, nitrate ($NO_3$) leaching, and $NH_3$ volatilization.

$N_2O$ is produced by nitrification and denitrification. Nitrification is modeled as a first order reaction of ammonium[16]. Denitrification is formulated as a three-step reduction of $NO_3$ to molecular nitrogen $N_2$, with nitrite ($NO_2$) and $N_2O$ as intermediates[17]. The denitrification rate is assumed proportional to the carbon dioxide production from the decomposition of soil organic matter[18] (SOM). Temperature and soil-water dependence of nitrification and denitrification are parameterized as described in Schmid et al.[14].

The emissions of $N_2O$ to the atmosphere are calculated with a resistance model. The total resistance is obtained from the diffusion coefficient of $N_2O$ in the main rooting zone and the aerodynamic resistances in the boundary layer (separately for the soil and the canopy).

Empirical parameters in the formulation of the N cycle are taken from independent sources in the literature. The overall time step of integration is set to 1 h, except for denitrification, which requires a time step of the order of 1 min. Some of the initial conditions (for instance, SOM) are determined through equilibrium simulations using the site-specific parameters. Management options relevant to the case study include cutting and the application of mineral fertilizers.

## PRODUCTION AND EMISSIONS OF $N_2O$: MEASUREMENTS AND SIMULATIONS

The basic results of the experiment carried out at Kerzersmoos are presented in Fig. 3.

Figure 3 reveals several key issues:

1. As expected, large emissions occur in the high N plot (Kerzers East) after the application of mineral N fertilizer. However, substantial emissions are observed on both plots 5 days after mowing. A possible explanation is the activation of a source of nitrate through mowing. Recalling that the sward is a mixture of grass and clover, it is conceivable that (a) nitrate is released after defoliation from protein-rich clover roots; or (b) denitrification is increased by Rhizobium within root nodules of clover as a result of changes in the symbiotic relationship; or further, (c) soil respiration is stimulated through increased availability of labile C from leaf and root litter and exudation after mowing. The effects of weather are discussed below under 3.
2. Emissions of $N_2O$ are roughly in phase with the soil concentrations of $N_2O$ at 2-cm depth and for the high N plot (Kerzers East), also with the concentrations at 5- and 25-cm depth. However, the correlation between emissions and concentrations at 5- and 25-cm depth in the low N plot (Kerzers West) is modest, implying that in both plots emissions are only determined by the concentrations in the very topsoil. This result is in line with the findings in Neftel et al.[7], which showed that the characteristic scale length for $N_2O$ in this soil is only of the order of 1 cm.

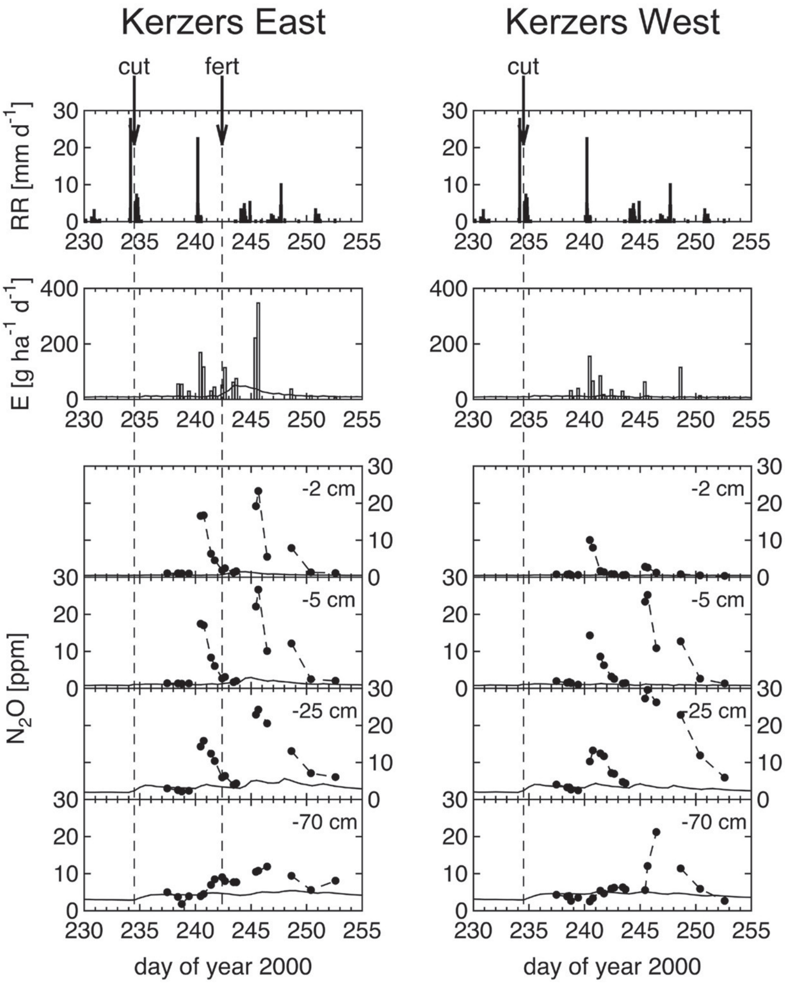

**FIGURE 3.** Time series (August 17th, day 230, to September 12th, day 255, 2000) of (top to bottom): (1) precipitation (RR); (2) observed (bars) and modeled (continuous line) $N_2O$ emissions (E); (3) observed (dots and dashed lines) and modeled (continuous lines) $N_2O$ concentrations in the soil at four different depths (2, 5, 25, and 70 cm). Mowing (cut) and fertilization (fert) are indicated by the arrows on top of the uppermost panel and by the vertical dashed lines. Left are the data referring to the high N-input plot (Kerzers East), right those referring to the low N-input plot (Kerzers West).

3. Production and emissions of $N_2O$ appear to be modulated by precipitation events (especially on day 240), which raise the soil water content of the topsoil to about 95% of its saturation level (not shown). Note the delay of 5 days between cutting and the first $N_2O$-emissions peak in the high N plot (Kerzers East), suggesting a time scale of this order of magnitude for either nitrate release from clover (see above) or for the holding capacity of nitrate in the topsoil.
4. Between days 245 and 250, large $N_2O$ concentrations are also observed at 70-cm depth, suggesting strong leaching of $NO_3$ due to almost continuous precipitation between days 243 and 249. Under these circumstances, denitrification becomes the dominant production mechanism for $N_2O$.
5. The model is not able to capture essential characteristics of $N_2O$ production in the soil, and therefore cannot correctly simulate $N_2O$ emissions. In particular: (a) the model catches the background concentrations of $N_2O$, but misses the peaks induced by precipitation, which means that (b) the calculated water fluxes are probably not accurate enough; (c) the model slightly overestimates the background emissions[19]; and, (d) the model largely fails to duplicate the effect of mowing.

## DISCUSSION

In the title, we asked to what extent can the exchange of gaseous N species be influenced actively, i.e., by management on a day-to-day basis. We now attempt to give an answer.

The results of our case study indicate that $N_2O$ emissions are triggered by management. In this specific instance, emissions induced by mowing are comparable to those brought about by the application of mineral fertilizers. However, the link between management and emissions is not a direct one, as the emissions are strongly modulated by precipitation on time scales ranging from 1 to 10 days. Therefore, active control of $N_2O$ emissions through management is only feasible provided that precipitation (and, in general, other environmental conditions) can be continuously monitored and quantitatively forecasted on time scales of the order of 1 week. If this is not the case, management will determine at most the order of magnitude of the emissions. Mitigation of the emissions of $N_2O$ and other gaseous N species at the operational level will then be possible only by strong interventions.

The ecosystem model used in this study is not yet in the position to reproduce individual events in the N cycle (production, transport, and release/uptake of gaseous N species). We have identified two problematic elements: (1) the omission of relevant processes in the N cycle, and (2) the simulation of the soil water fluxes (rather than soil water content). In addition, we have to be aware of the simplistic description of the distribution of soil organic matter. SOM is assumed constant over the whole soil profile, although here, as in most soil types, vertical gradients are probably large. In the model, denitrification is assumed proportional to the turnover of SOM, which means that the potential for denitrification is also equally distributed over the whole soil. This might lead to systematic errors not only in the soil concentrations, but also in the emissions of $N_2O$.

As noted in Schmid et al.[19], and partially seen in our results, PaSim has a slight tendency to overestimate the background emissions of $N_2O$, but underestimates several of the individual emissions peaks. This must be taken into account when looking at annual average emissions. It is possible that ecosystem models generally produce annual emissions of the correct order of magnitude, despite severe inaccuracies in the dynamics.

PaSim has recently been extended[20] to allow for a more detailed treatment of the exchange of $NH_3$. On the other hand, NO (a by-product of denitrification) is not yet considered in the model. This could represent an additional source of errors for the simulation of $N_2O$. It is recommended that future generations of ecosystem models (PaSim and others) attempt an integrated account of the whole N cycling, paying the same amount of attention to each individual component. Efforts to strengthen only selected modules may not improve the overall performance of the model.

## ACKNOWLEDGEMENTS

We thank Dr. Martin Schmid and Dr. Marcel Riedo for their help in running PaSim, and two reviewers for instructive comments.

| | | |
|---|---|---|
| Received: | July | 16, 2001 |
| Revised: | November | 6, 2001 |
| Accepted: | November | 9, 2001 |
| Published: | November | 30, 2001 |
